# Distinct brain network organizations between club players and novices under different difficulty levels

**DOI:** 10.1002/brb3.3488

**Published:** 2024-04-19

**Authors:** Chantat Leong, Zhiying Zhao, Zhen Yuan, Bin Liu

**Affiliations:** ^1^ Centre for Cognitive and Brain Sciences University of Macau Macau SAR China; ^2^ Faculty of Health Sciences University of Macau Macau SAR China; ^3^ Department of Emergency Zhujiang Hospital, Southern Medical University Guangzhou China

**Keywords:** chunk memory, difficulty, expertise, functional connectivity, topological characteristic

## Abstract

**Significant:**

Chunk memory is one of the essential cognitive functions for high‐expertise (HE) player to make efficient decisions. However, it remains unknown how the neural mechanisms of chunk memory processes mediate or alter chess players’ performance when facing different opponents.

**Aim:**

This study aimed at inspecting the significant brain networks associated with chunk memory, which would vary between club players and novices.

**Approach:**

Functional networks and topological features of 20 club players (HE) and 20 novice players (LE) were compared at different levels of difficulty by means of functional near‐infrared spectroscopy.

**Results:**

Behavioral performance indicated that the club player group was unaffected by differences in difficulty. Furthermore, the club player group demonstrated functional connectivity among the dorsolateral prefrontal cortex, the frontopolar cortex, the supramarginal gyrus, and the subcentral gyrus, as well as higher clustering coefficients and lower path lengths in the high‐difficulty task.

**Conclusions:**

The club player group illustrated significant frontal–parietal functional connectivity patterns and topological characteristics, suggesting enhanced chunking processes for improved chess performance.

## Graphical abstract

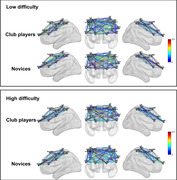

## 1 INTRODUCTION

The theory of chunks (Chase & Simon, 1973) was first proposed to inspect the cognitive processes of chess players. According to the chunk theory, when a pattern is recognized, the chunking process involves an interaction between recognition and search processes. This interaction leads to an increase in search depth, resulting in a quicker decision‐making process (Gobet, 1997). Through extensive skill training, experts can store their experiences as perceptual chunks in long‐term memory. This allows for more efficient search processes and more sophisticated evaluation functions to assess decisions (Charness, 1981; Connors et al., 2011; Lassiter, 2000). In particular, the cognitive patterns for the chess experts can be broken down into two components: segmentation and concatenation. In the chunking process, segmentation refers to the spontaneous division of complex scenes or continuous information into thematic chunks when individuals recognize perceptual patterns. By contrast, concatenation involves combining short chunks into longer segments and enhancing decision‐making effectiveness in complex environments (Sakai et al., 2003; Verwey & Eikelboom, 2003; Verwey, 1996, 2001).

To date, neuroimaging techniques have been used to inspect the cognitive neural mechanism underlying decision‐making in playing chess. It was discovered that distinctive neural activation patterns were identified in chess experts as compared to those of novices during chess‐related tasks (Campitelli et al., 2007). In particular, functional magnetic resonance imaging studies demonstrated the interplay between the frontal and parietal regions in experts, involving a chunking process during the chess task (Bilalić et al., 2010; Gobet & Charness, 2018; Schubotz et al., 2012; Song et al., 2020; Song et al., 2022). More importantly, previous studies also illustrated that distinct brain regions were involved in the segmentation and concatenation processes within the chunking process. For example, frontal cortex activation is central to the segmentation of the chunking action (Zalla et al., 2001), whereas the supramarginal gyrus (SMG) and inferior frontal gyrus (IFG) are involved in controlling the chunking rate during segmentation (Leong et al., 2023; Park et al., 2015; Rimmele et al., 2021). In the concatenation process, parietal areas, including the supramarginal and postcentral gyri, are implicated in visual recognition, memory integration, and concatenation within the chunking process (Carlomagno et al., 2022; Tomasi et al., 2007).

In addition, graph theory serves as a tool for studying the functional networks of brain organization, particularly small‐world features (Bullmore & Sporns, 2009; He & Evans, 2010). It was widely recognized that elevated small‐world characteristics, clustering coefficients, and shorter path lengths signify heightened information transfer efficiency within functional networks (Achard & Bullmore, 2007; Sporns et al., 2004). Interestingly, previous findings demonstrated a positive association between expertise and topological properties in Chinese chess experts, indicating a connection between functional networks and the chunking process (Duan et al., 2014; Li et al., 2009).

Further, the stduy was discovered that paradigm difficulty showed the relationship with cognitive performance and problem‐solving skill differences among experts. Previous electroencephalogram studies suggested that experts can reduce cognitive load and enhance neural efficiency. However, when pressure increases, theta power rises for experts while decreasing for novices (Fuentes‐García et al., 2020). Similarly, a heart rate variability (HRV) study demonstrated that experts were able to adjust their autonomic modulation (HRV) in tasks with higher difficulty (Fuentes‐García et al., 2019). These findings indicate that the neural and physiological activities of chess experts respond differently to changes in task difficulty as compared to novices. However, little is known about the impact of task difficulty on the topological properties of brain networks in playing chess. In this study, we hypothesize that, (1) experienced and inexperienced players will exhibit different brain network topological properties with the same difficulty levels, and (2) as task difficulty changes, experienced and inexperienced players might show distinct patterns of topological changes in brain networks associated with chunk memory processes, particularly in frontal and parietal networks.

To test the hypothesis, functional near‐infrared spectroscopy (fNIRS) neuroimaging was carried out to inspect the cognitive neural mechanism underlying playing chess. In particular, we demonstrated the difference in brain activation pattern between club players and novices during chess tasks with various difficulty levels. Graph theory analysis (Bullmore & Sporns, 2009; Stam & Reijneveld, 2007) was used to examine topological properties across different difficulty levels, successfully accessing the small‐world network characteristics and network efficiency of chunk memory process.

## 2 METHODS

### 2.1 Participant

Twenty chess players were invited and considered the club player group (high‐expertise [HE]). The average age of the club player group was 22.8 ± 2.53. The expertise level of chess players was determined using the ELO rating system, developed by Elo (1978) and applied by the Federation Internationale des Echecs (FIDE). The Elo rating of the chess player group in this study was 1501.85 ± 77.37. Another 20 players were recruited as the novice group (low‐expertise [LE]). The average age of the novice group was 21.75 ± 1.17 years, and ELO was Elo: 1092.85 ± 105.85. Definitions of club players and novices are based on the HIARCS Chess Explorer website (https://www.hiarcs.com/hce‐manual/pc/Eloratings.html). In addition, the club player group requires 2 or more years of experience, and the novice group requires up to 3 months of experience. Neither group was physically or mentally sick. All participants were asked to fill out a consent form before the experiment. The experimental protocol was approved by the Institutional Review Board of the University of Macau [BSERE21‐APP019‐ICI‐01].

### 2.2 Design and procedure

The chess games interface was developed by Moore (2022) using Stockfish 5, a widely used open‐source (GPL license) chess engine. The Stockfish engine serves as a valuable tool in chess practice, simulating human tactical responses and providing an effective simulation of the chess environment (Regan et al., 2014). Participants were tasked with facing two opponents of differing skill levels (Elo 1350 and Elo 1800). The task had a time limit of 3 min, requiring players to complete a minimum of 15 moves within this timeframe.

**FIGURE 1:**
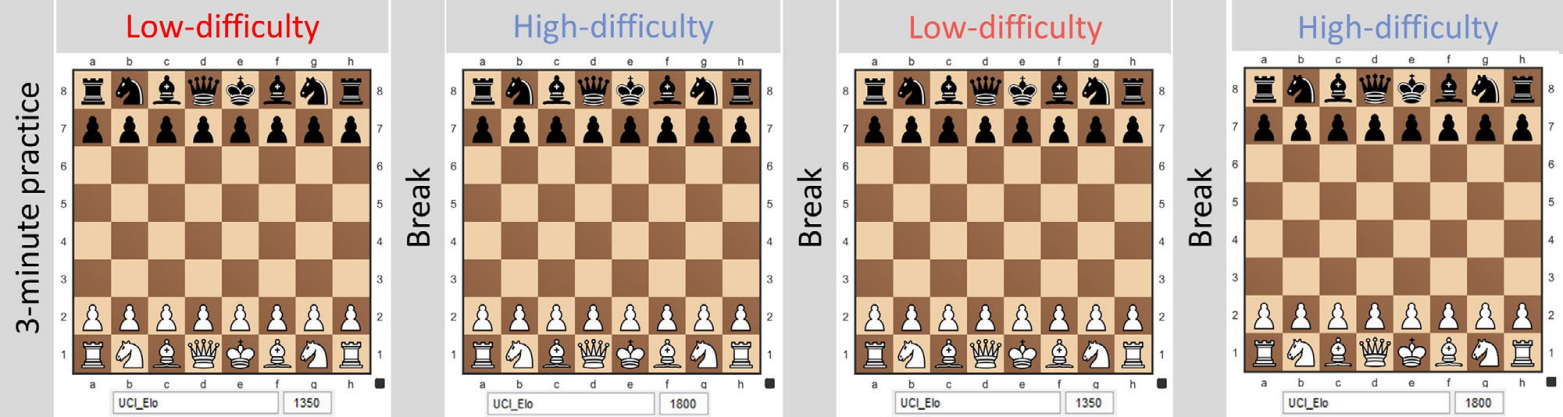
Paradigm of chess difficulty tasks. Participants played two 3‐min games against low‐difficulty (LD) and high‐difficulty (HD) opponents. There was a 3‐min break between games. The order of the games was randomly assigned. This figure illustrates one possible assignment of chess tasks.

Participants were seated 55 cm away from the display. Prior to the formal test, participants engaged in a practice session involving a 3‐min game set at a low level of difficulty. The formal test series was organized as depicted in Figure 1. Participants played a 3‐min game against a 1350 Elo opponent (low‐difficulty, LD), followed by two 3‐min games against 1800 Elo opponents (high‐difficulty, HD), and finally another 3‐min game against a 1350 Elo opponent, or vice versa. Player had 2‐min break between each game. The order of the games was randomly assigned to prevent the warm‐up effect (Elsworthy et al., 2013) and anticipatory effect (Tucker, 2009).

### 2.3 Behavioral data analysis

During the chess tasks, inaccuracy rates, which indicate the mistakes made during chess games, were computed using the Stockfish engine after each game. Task performance across different levels of chess expertise and task difficulty was compared using a two‐way analysis of variance (ANOVA) test. The significance threshold for statistical significance was set at *p* < .05.

### 2.4 fNIRS measures and pre‐process

The imaging data were captured using a continuous wave fNIRS system (CW6 fNIRS). This system employed 16 light sources and 23 detectors with 2 wavelengths (690 and 830 nm) to record both oxyhemoglobin (oxy‐Hb) and deoxyhemoglobin (deoxy‐Hb) signals in the human brain, all sampled at a frequency of 50 Hz. The configuration comprised 55 measurement channels, each spanning 30 mm, and covering frontal, motor, and parietal regions. The three‐dimensional (3D) positions of the light sources and detectors were established using a 3D digitizer, which recorded the reference channels (cz, nz, lz, lpa, and rpa) and the target channels (55 channels). Each channel was then compared and estimated by NIRSKIT (Hou et al., 2021) in terms of 3D digitizer and coordinates on a standard template for the brain to derive Montreal Neurological Institute (MNI) coordinates for each channel, which facilitated the localization of the corresponding brain regions.

The fNIRS raw data were preprocessed and transferred to optical density (OD) values by using HOMER3 (Huppert et al., 2009). A band‐pass filter (0.1–0.01 Hz) was applied to mitigate physiological noise, encompassing heartbeat and respiration artifacts. Subsequently, the spline‐SG hybrid method algorithm (Jahani et al., 2018) and the wavelet‐based method algorithm (Cui et al., 2010) were applied to eliminate head motion and eye movement artifacts. Third, OD values were further filtered through the skin blood flow by using principal component analysis (Zhang et al., 2005). Fourth, the oxy‐Hb and deoxy‐Hb concentrations were generated using filtered OD values after normalized unit variance (Ding et al., 2013; Hu et al., 2018). In particular, the modified Beer–Lambert law was applied, and the differential path length factor of the infrared wavelength rate was set to 6.0, which was an adequate value for the present study (Scholkmann & Wolf, 2013). Finally, the general linear model was used to estimate the hemodynamic response function for each player. The oxy‐Hb and deoxy‐Hb concentration data were converted to beta values (*β* values), which represent the time series of the hemodynamic response (Hu et al., 2010; Lindquist et al., 2009). For the present study, the oxy‐Hb signals were the focus, as they demonstrate heightened sensitivity to changes in cerebral blood flow (Fu et al., 2014).

### 2.5 Functional connectivity network analysis

**FIGURE 2:**
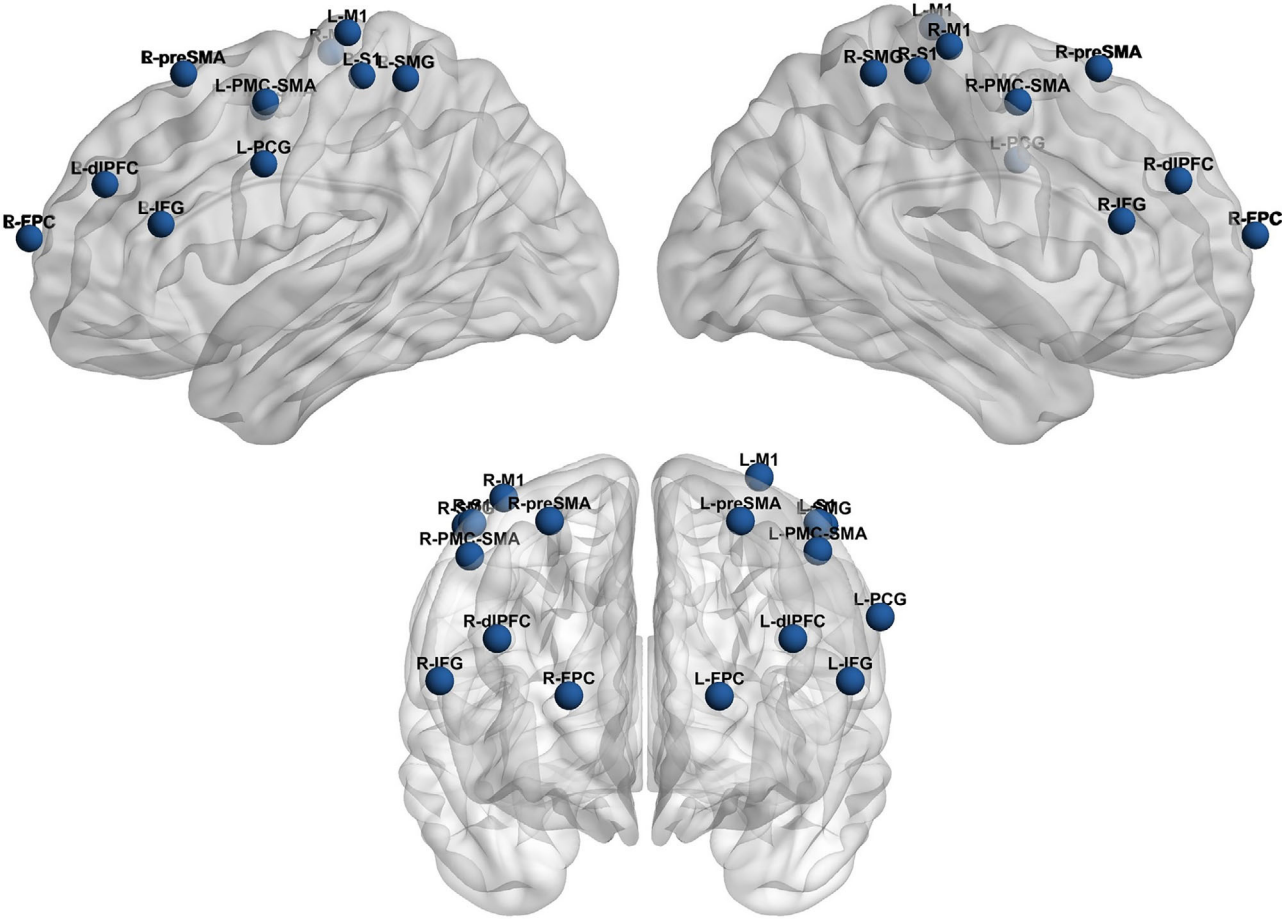
The three‐dimensional (3D) Montreal Neurological Institute (MNI) coordinates of region of interest (ROI). Front and side view layouts of 17 ROIs. The coordinates of ROIs are shown as blue spheres.

In this study, in order to construct the nodes of the functional network, we calculated the highest similarity values of the brain regions represented in all channels in order to categorize 17 regions of interest (ROIs), as shown in Table 1. Those spatial positions corresponding to the channels of each ROI are averaged and considered ROI nodes. To determine the edges among nodes, for each subject, the representational beta values in each node were obtained by averaging channels’ beta values across all ROI (Figure 2). Pearson's correlation was then applied to construct a correlation matrix (17 × 17 functional connectivity matrix) for all participants, and the Fisher *z*‐transform was further calculated to enhance the normality of the matrix (Duan et al., 2014; Leong et al., 2023; Liao et al., 2010; Liu et al., 2008). ANOVA tests were further applied to compare the functional connectivity matrix in difficulty and chess ability. Here, we used a false discovery rate (FDR) correction and controlled for *p* < .05 to minimize type 1 error. FDR is a statistical approach employed to correct for multiple comparisons, addressing the issue of random events that erroneously appear significant (Storey, 2002).

**TABLE 1:** Estimated Montreal Neurological Institute (MNI) coordinates for the channel.

Region	Brodmann area	Category	Hemisphere	Channel	MNI coordinates		
FPC	10	Frontal	Right	1,3	21.15	69.65	12.80
			Left	29,31	−20.65	69.65	12.65
DLPFC	9		Right	2,8,10,11,12	41.06	47.28	28.66
			Left	30,36,38,39	−41.06	47.28	28.66
IFG	45		Right	4,6,7,9	57.00	30.68	16.90
			Left	32,34,35,37	−57.00	30.68	16.90
Pre‐SMA	8	Parietal‐motor	Right	13,19	26.50	24.00	61.35
			Left	41,47	−26.50	24.00	61.35
PMC‐SMA	6		Right	5,14,15,18,20,21	48.67	0.40	51.55
			Left	33,45,46,48	−48.00	−0.26	52.98
SMG	40		Right	24	49.70	−41.70	60.00
			Left	51	−49.70	−41.70	60.00
SCG	43	Left	42,43	−65.30	0.30	34.70
M1	4	Motor	Right	17,25,26	39.23	−19.57	67.77
			Left	52,53	−31.70	−24.50	73.50
S1	1		Right	16,22,23,27,28	48.00	−28.74	60.74
			Left	44,49,50,54,55	−48.00	−28.74	60.74

Abbreviations: FPC, frontopolar cortex; DLPFC, dorsolateral prefrontal cortex; IFG, inferior frontal gyrus; Pre‐SMA, pre‐supplementary motor area; PMC‐SMA, Pre‐Motor Cortex‐Supplementary Motor Area; SMG, supramarginal gyrus; M1, right motor area: SCG, subcentral gyrus.

In addition, we aimed to examine the relationship between chunk memory in terms of both behavioral and neural dimensions. Subsequently, we investigated whether the increases in connectivity in various regions across subjects correlated with chess move accuracy. For each condition, we calculated the correlation coefficients between functional connectivity and behavioral performance in four conditions and corrected for FDR (*p* < .05).

### 2.6 Graph theory analysis

To investigate the network characteristics of functional connectivity, the GRETNA toolbox was used to construct and investigate the topological properties of brain networks (Wang et al., 2015). In graph theory, nodes are brain regions, and edges serve as connections. In the present study, each subject's correlation matrix was computed by thresholding (*T*) and determined by sparsity (*S*). Sparsity ensures that all networks have the same number of edges and nodes and eliminates within‐group differences in connected networks (Achard & Bullmore, 2007; Xu et al., 2020). In our study, we set the threshold range to 0.01 < *S* < 0.50 with an interval of 0.05 for each weighted matrix. The threshold range was adopted based on the criterion of small‐worldness scalar that guaranteed small‐worldness estimation. The connectivity matrix was generated in binary (global efficiency and local efficiency), which computed network characteristics, including the clustering coefficient, the average feature cluster coefficients (*C_p_*), path length (*L_p_*), network coefficients (*E_g_*), and small‐worldness (*σ*).

The *C_g_* is the average cluster coefficients across the nodes, *C_i_* is the cluster coefficients of coefficient node, it represents the number of triangles, and *K_i_* is the number of connections (Rubinov & Sporns, 2010; Watts & Strogatz, 1998; Xu et al., 2020):

$$\;C_{p}=\;\frac{1}{n}\sum_{i\in N}\;C_{i}=\;\frac{1}{n}\sum_{i\in N}\frac{2t_{i}}{K_{i}\left(K_{i}-1\right)}$$

The characteristic path length (*L_p_*) measures the overall network efficiency (Watts & Strogatz, 1998), which is defined as the sum of edge lengths between two nodes. In GRETNA, *L_p_* represents the shortest weighted path between network nodes (*d_ij_*), which is measured as the harmonic mean of pairs to avoid network disconnections (Newman, 2003; Rubinov & Sporns, 2010):

$$L=\frac{1}{1/N\left(N-1\right)\sum_{i\;=\;1}^{N}\sum_{j\neq 1}^{N}1/d_{ij}}$$

Small‐world characteristics were proposed by Watts and Strogatz (1998): A real world would be considered small‐world when it had higher *C_p_* and *L_p_* similarity than a random network. $C_{rand}$ and $L_{rand}$ were the 100 matched random networks’ *C_p_* and *L_p_* that had the same as the real network. The network was considered a small‐world characteristic when small‐worldness (*σ*) was equal to or larger than 1 (Rubinov & Sporns, 2010):

$$\sigma \;=\;\frac{C/C_{rand}}{L/L_{rand}}$$

The global efficiency (*E_g_*) is the inverse of *L_p_*, which measure the functional aggregation in the network (Achard & Bullmore, 2007):

$$E_{g}=\;\frac{1}{1/N\left(N-1\right)\sum_{i\neq j\in N}\frac{1}{L_{ij}}}$$

To address the difference in the difficulty of the brain network between club players and novices, a two‐way ANOVA test was utilized to compare the differences of the functional networks and topological properties, including *L_p_*, *E_g_*, and *σ*, across all conditions. We adopted the area under the curve over the range of *S*, which can provide the summarized characterization of brain networks (Achard & Bullmore, 2007; He et al., 2009). The significant level of each topological characteristic analysis was set to *p* < .05 with FDR correction (Storey, 2002).

## 3 RESULTS

### 3.1 Behavioral result

The analysis revealed a significant difference between the HE and LE groups (*p* = 2.41e − 5, and *F* = 20.24) after performing a two‐way ANOVA on the mistake rates. In addition, a significant interaction effect was found across the four conditions (*p* = .0361, *F*(1, 76) = 4.551), suggesting that mistake rates in the HE group were not affected by differences in difficulty compared to the LE group.

### 3.2 Functional connectivity and correlation analysis results

**FIGURE 3:**
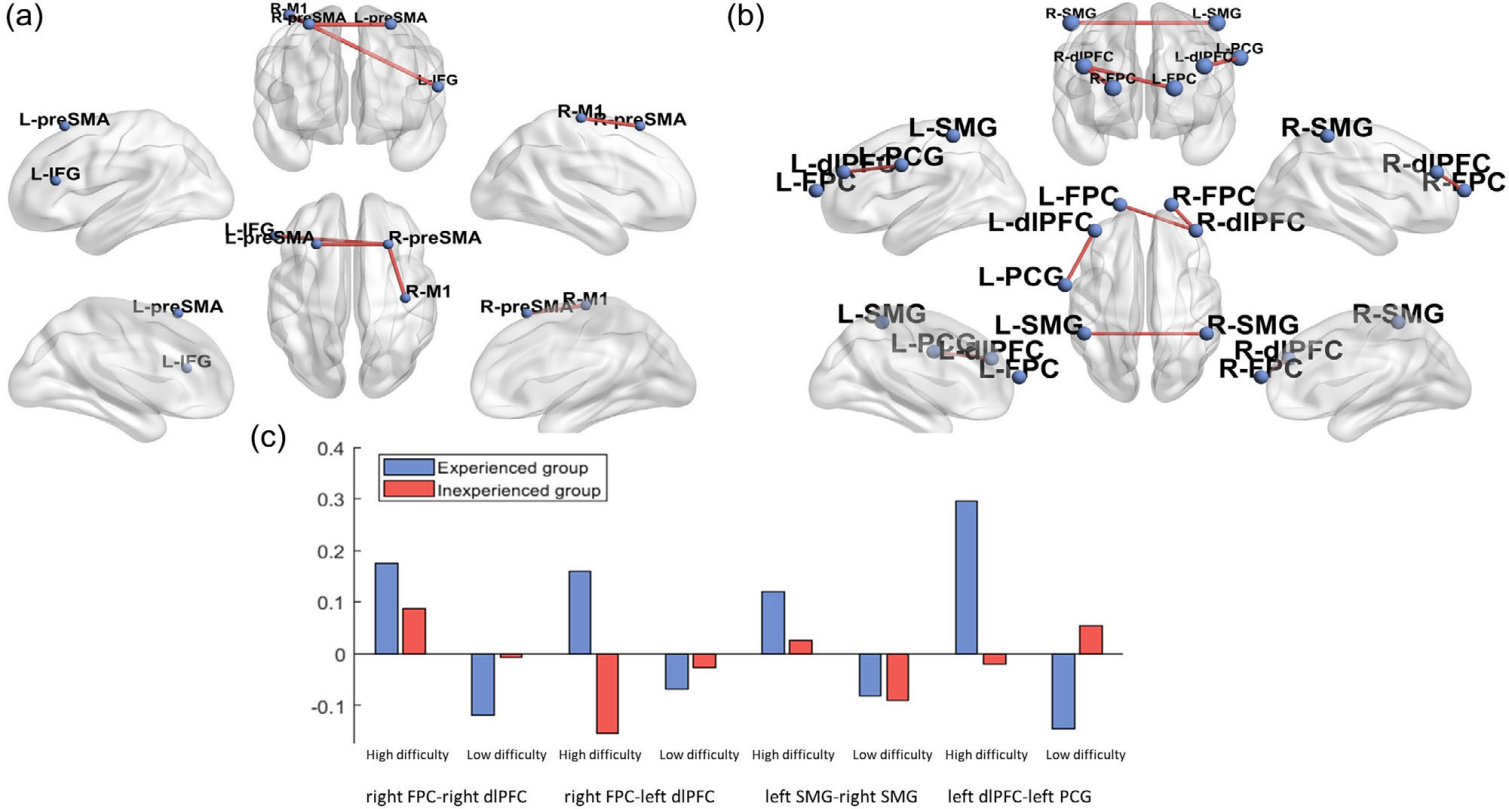
Brain network differences across expertise and difficulty: (a) comparison between high‐expertise (HE) and low‐expertise (LE) groups; (b) interaction across difficulty and chess expertise. Nodes (blue spheres) are positioned according to the 17 region of interest (ROI) coordinates, and red edges indicate results where significant connections are drawn (panels a–c) for multiple comparisons across chess expertise × game difficulty. Red indicates the inexperienced group, and blue indicates the experienced group (panel c).

A 2 (HE vs. LE) × 2 (HD vs. LD) ANOVA was conducted on the measure of connectivity networks as well. After excluding *p* > .05 with FDR‐corrected functional connectivity, there were six connections in the comparison between HE and LE chess groups and three connections between HD and LD opponents. Descriptive statistics for connections are illustrated in Figure 3. Specifically, for the HE group, significant connections were demonstrated in pre‐supplementary motor area (pre‐SMA), left IFG, and right motor area (M1) for the HD task as compared to the LD, including right pre‐SMG‐right M1 (*p* = .047, *F*(1, 76) = 4.07), right pre‐SMG‐left pre‐SMA (*p* = .0073, *F*(1, 76) = 8.90), and right pre‐SMA‐left IFG (*p* = .032, *F*(1, 76) = 8.90). However, there was no significant connection between the HD and LD conditions. Additionally, multiple comparisons further showed that in the HD condition, the HE group demonstrated greater functional connectivity compared to the LE group in the following areas: frontopolar cortex (FPC), dorsolateral prefrontal cortex (dlPFC), SMG, and left subcentral gyrus (SCG) as compared to the LE group, including right FPC‐right dlPFC (*p* = .026, *F*(1, 76) = 5.12), right dlPFC‐left FPC (*p* = .030, *F*(1, 76) = 4.91), right SMG‐left SMG (*p* = .045, *F*(1, 76) = 4.15), and left dlPFC‐left SCG (*p* = .0047, *F*(1, 76) = 8.47).

To investigate the relationship between connectivity changes and mistake rates, we calculated the correlation coefficient across functional connectivity. The results were in the LE group with an HD task (Table 2), and the correlation exceeded the chance for right DLPFC‐left FPC (*p* = .0483, *r* = −.447). In the HE group with LD task, the correlation was significant in right DLPFC‐left DLPFC (*p* = .0544, *r* = −.454). In the HE group with HD task, the correlation exceeded the chance for right DLPFC‐left FPC (*p* = .0388, *r* = −.465) and left DLPFC‐left SCG (*p* = .0012, *r* = −.67).

**TABLE 2:** Correlation between behavioral responses and brain networks in the chess task.

Condition	Comparison	*p*‐Value	*r*
High‐difficulty × low‐expertise	Right DLPFC‐left FPC	.0483	−.447
Low‐difficulty × high‐expertise	Right DLPFC‐left dlPFC	.0454	−.454
High‐difficulty × high‐expertise	Right DLPFC‐left FPC	.0388	−.465
	Left DLPFC‐left SCG	.0012	−.67

Abbreviations: DLPFC, dorsolateral prefrontal cortex; FPC, frontopolar cortex; SCG, subcentral gyrus.

### 3.3 Increased network characteristics across different conditions

**FIGURE 4:**
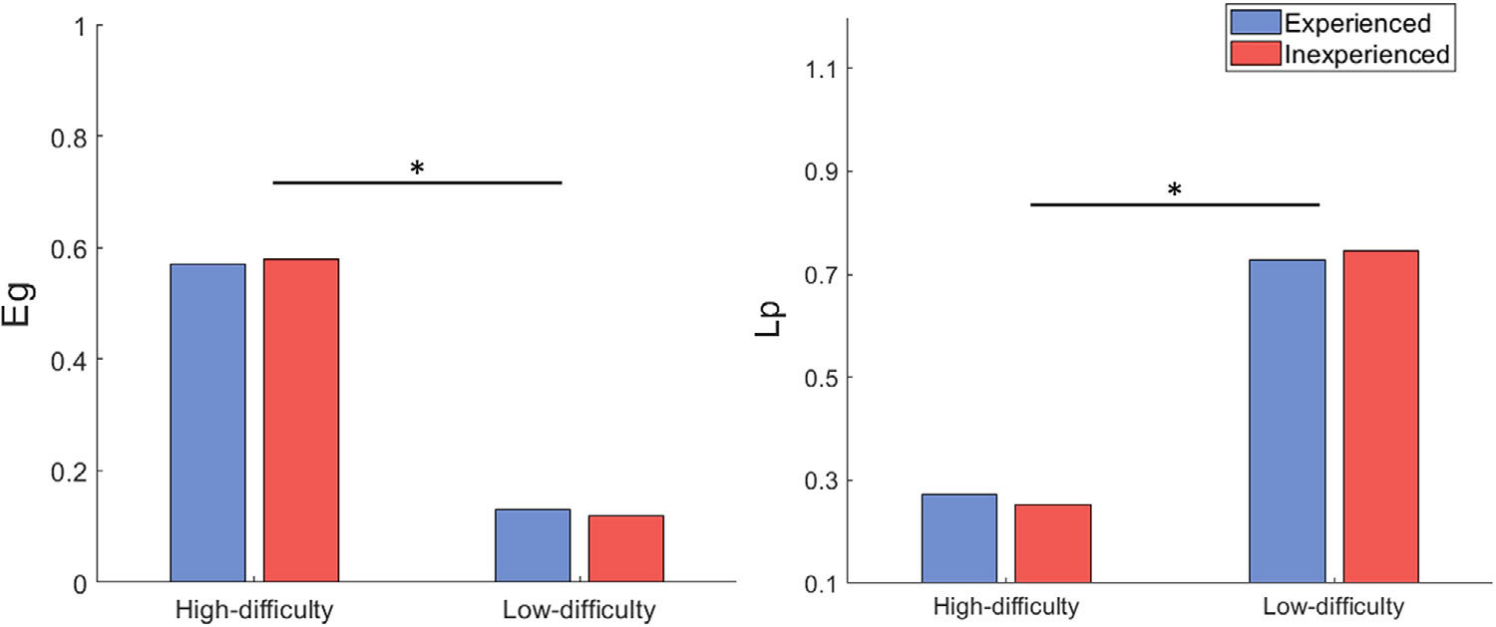
Group differences in *E_g_* and *L_p_* of functional connectivity networks. Red indicates the inexperienced group, blue indicates experienced group, and the asterisk (*) indicates a significance level of *p* < .05.

The average path length (*L_p_*), global efficiency (*E_g_*), and the measure of small‐worldness (*σ*) were, respectively, calculated across four conditions (HD, LD, HE, and LE). As the *σ* of connectivity networks for all conditions was larger than 1 as compared to that of random paired networks, the connectivity networks for all conditions manifested small‐world properties (Figure 4). More importantly, the same two‐way ANOVA was performed on the average path length (*L_p_*), global efficiency (*E_g_*), and small‐world metric (*σ*). To better interpret the functional connectivity patterns in the graph‐theoretic analysis, chess players demonstrated significantly larger *E_g_* (*p* = .028, *F*(1, 76) = 5.05) and shorter *L_p_* (*p* = .037, *F*(1, 76) = 5.05) in the HD task than in the LD task when the threshold *T* ranged between 0.2 and 0.3. Yet, there were no significant effects on small‐world properties (*σ*) for chess players facing either HD or LD opponents. In addition, no differences were shown in *E_g_*, *L_p_*, and *σ* for the HE and LE conditions.

## 4 DISCUSSION

In the present study, we compared the effects of expertise on behavioral performance and functional connectivity when facing different difficulties. To our knowledge, this is the first study to investigate the chunk memory in functional connectivity and network characteristics across (chess expertise × game difficulty) four conditions.

### 4.1 Behavioral performance

According to our behavioral findings, the HE group exhibited superior performance compared to the LE group. This observation suggests that the chunk memory process supports HE players in making more effective chess moves. In addition, within the HE group, changes in task difficulty did not significantly impact chess game performance, unlike the LE group. This finding implies that club player group possesses a distinct chunk memory process that enables them to maintain a high level of chess move quality even in challenging, HD scenarios.

### 4.2 Functional connectivity difference between high‐expertise and low‐expertise groups

In comparison between HE and LE chess players under different difficulties, the present study showed functional connectivity differences in prefrontal, motor, and regions, including the IFG, M1, and pre‐SMA. In line with the results of previous studies, this study found that the chunking behavior of HE players was supported by the frontal network more than that of novice players (Bo et al., 2009; Pammi et al., 2012; Verwey, 2010; Verwey et al., 2011). When frontal networks are activated, subjects are able to spontaneously segment information into chunks (Pammi et al., 2012; Wymbs et al., 2012) and evoke the best next move or the series of moves (Wan et al., 2011). In the present study, the HE group showed significant connections in IFG and pre‐SMA compared to the LE group, which contributed to the process of segmentation of chunks of memory. Segmentation refers to the search, segments, and recall of specific chunks in the recognition of previously HE perceptual patterns (Connors et al., 2011; Leong et al., 2023).

Specifically, our functional connectivity analyses yielded the correlation of difficulty with connectivity between predominantly pre‐SMA and left IFG, which are important in the search and recognition process (Lissek & Tegenthoff, 2021). Previous studies have reported functional networks between the pre‐SMA, M1, and during visual recognition to motor execution. Functional networks between the visual cortex and the prefrontal cortex, including the pre‐SMA and left IFG, are thought to mediate attentional and executive controls (Berken et al., 2016; Coderre et al., 2016). In detail, during the search and memory recall process, pre‐SMA exerts top–down influences on visual attention and recognition (Vossel et al., 2012) and exhibits sustained delay activity during the recognition and memory recall process (Offen et al., 2010). However, the left IFG is thought to mediate renewal and selection among competing response options (Budhani et al., 2007; Lissek & Tegenthoff, 2021; Zhang et al., 2004). Our results suggest that, consistent with previous findings, the segmentation process differs among HE groups. Such differences in functional connectivity patterns help support recognition and memory recall, including the detection and correction of erroneous movements.

### 4.3 Functional connectivity in the HE group with high‐difficulty task

Our interaction results revealed significant connectivity results in the HE group with the HD task, including right FPC‐right dlPFC, right dlPFC‐left FPC, right SMG‐left SMG, and left dlPFC‐left SCG. Previous studies suggest that the chunking process associated with expertise in frontal–parietal regions may be activated during more complex or difficult tasks (Bartlett et al., 2013). The frontal–parietal regions can form interacting memory systems, which contribute to segmentation and accelerate the process of chunk memory through repeated practice (Rimmele et al., 2021). FPC is associated with the application of cognitive resources. Our findings are consistent with previous studies showing that highly skilled groups (experts) exhibit significant associations in FPC, which suggests that cognitive resources are effectively moderated in highly skilled groups (Laureiro‐Martínez et al., 2014; Lu & Yuan, 2015; Zarate & Zatorre, 2008). Besides, our findings on the connection between the right DLPFC and the left FPC show a correlation between them and behavioral performance in HD tasks, which further confirmed that FPC plays an important role in the decision‐making process in chess game. When faced with stressful situations, such as difficult tasks, the FPC participates in allocating cognitive resources and encoding valid information to achieve optimal moves (Boorman et al., 2011; Mansouri et al., 2017).

DLPFC and SMG are associated with the chunking process and play an important role in the segmentation process. In the segmentation process, both DLPFC and left SMG are associated with object identification and retrieval action (Kellenbach et al., 2005) and contribute to subjects selecting the necessary items from memory to respond (Rowe et al., 2000; Rowe & Passingham, 2001). Reverberi et al. (2005) have revealed that in higher order neural networks (decision planning and making), the dlPFC participates in top–down modulation of the network. The right DLPFC contributes to information storage, manipulation, or management (Pochon et al., 2001; Volle et al., 2005). In the memory recall process, the right DLPFC is important for memory information recall and error detection (Fletcher, 2001; Navarro‐Cebrian et al., 2016). Right DLPFC has suggested that it plays a role in the segmentation of chunk memory (Hamidi et al., 2009). Left SMG contributes to pattern relationship recognition and the information retrieval process (Kellenbach et al., 2003) and manifests a high correlation in successful segmentation (Park et al., 2015). The activation of the right dlPFC and the left SMG rises when club players are confronted with a familiar environment, implying that the club players can mediate better segmentation by allocating cognitive resources. The left dlPFC is considered to alter memory during the learning phase, such as updating, tracking, or integrating memory information (Leong et al., 2023; Verwey et al., 2015), whereas the left SMG is suggested to regulate the process of recording and retrieving chunk memory (Rimmele et al., 2021).

Aside from frontal networks, SCG was also involved in the chunking process. SCG has been suggested to be associated with motor action in the recognition task (Kato & Izumiyama, 2015; Porro et al., 1996) and involved in memory processes (Mainy et al., 2007). In the HE group, SCG recruits a larger brain network to complete higher performance (Fernández‐Rubio et al., 2022). The engagement of SCG is attributable to the spatial representational pattern manipulation and separates the pattern into individual components and regrouping in meaningful ways, which indicates the connection of SCG is important in the concatenation of the chunk memory process (Huang et al., 2015; Luo et al., 2006; Wu et al., 2013). In addition, our correlation results showed that the HE chess game performance on HD tasks was associated with the functional connectivity in DLPFC and SCG. This suggests that both the DLPFC and the SCG are involved in the regulation of chunk memory processes on the dimensions of chess expertise and chess game difficulty. Thus, our results suggest that club players differ in their chunking process under HD conditions, thus maintaining high‐quality moves in chess games.

Overall, based on differences in functional connectivity patterns, our findings suggest that chunking memory processes differ when faced with more difficult opponents. In the face of HD tasks, through differences in the frontal–parietal network, our findings suggest that club players can manipulate more cognitive resources in the DLPFC and SMG for segmentation processes and recall memory information from chunks. This difference in functional connectivity patterns helps support recognition and memory recall, including the detection and correction of erroneous movements. During concatenation, SCG connections further reorganize procedural chunks and integrate them into larger chunks, resulting in more efficient decision‐making behavior that is transferred to the final motor action.

### 4.4 Enhanced topological properties in high‐difficulty task

According to previous neuroimaging studies, high levels of cluster coefficients and short path lengths in functional networks imply that functional networks have a high level of small‐world topology (Achard & Bullmore, 2007; Bassett & Bullmore, 2006). Consistent with previous findings, our result showed the functional network exhibited small‐world properties (*σ* was larger than 1) in all four conditions, which implies that brain networks have efficient information transfer capabilities (Achard & Bullmore, 2007; Duan et al., 2014; Sporns et al., 2004). It is suggested that topological properties are positively associated with cognitive performance. People with short path length have higher intelligence quotient scores (Van Den Heuvel et al., 2009). Similar results have been reported for the association between brain networks and cognitive performance (Duan et al., 2014).

In the present study, although all conditions manifested small‐world properties, the topological properties of cluster efficiency and shortest path length were significant and difficult comparisons. The increase in cluster coefficient and shorter path length implies a stronger connectedness of the brain networks when facing HD opponents. The clustering coefficients correspond to the interconnectedness in the network, showing the influence of functional connectivity between different difficulties. According to the functional connectivity analysis in the previous section, chess players demonstrated different connectivity patterns in the chunk memory process, including DLPFC, FPC, SMG, and SCG when facing an HD opponent. It is indicated that in HD chess games, these connectivity patterns are more efficient in transferring cognitive information in the segmentation and concatenation process as compared to LD chess games. As a result, effective and accurate chess moves are produced in easier conditions.

## 5 LIMITATIONS AND FUTURE PERSPECTIVES

There are some limitations to this study that need to be noted. First of all, there are some differences in personal perceptions of the difficulty of the game. However, considering the current research, the definition and effect of individual differences are still unclear. We sought to match the difficulty and playing level of club players with the simplicity and playing level of novices. At the same time, due to the limited number of experts recruited, we have lowered the recruitment requirements and recruited club player level at the end. According to ELO ratings, there is still a significant difference in the level of expertise between club players and novices. From another perspective, our results reveal differences in the functional reorganization of the brain between chess players (on average) and novices. This finding may convey a more significant message: The game of chess has a substantial impact on the regulation of brain connectivity, even at an average level. Moreover, due to the limitations of the paradigm and near‐infrared spectroscopy, the present study was limited to studying only the frontal–parietal cortical region. Future research can improve the paradigm so that it can be adapted to MRI as well as better cater to individual differences.

## 6 CONCLUSION

We conducted an investigation into the functional connectivity and network topological properties of novices and club players engaged in two distinct tasks of varying difficulty. Our findings showcase that the connectivity networks within the IFG, premotor cortex‐supplementary motor area, and pre‐SMA exhibit notable differences in the club player group when contrasted with the novice group. This discrepancy points toward functional networks that are intricately linked to the effects of segmentation processes, encompassing perceptual pattern recognition, memory information retrieval, and execution.

Upon comparing club players and novices, our results underscore distinct functional connectivity patterns in the FPC, dorsal frontoparietal cortex (DFPFC), SMG, and SCG within the HD task context. This suggests a compelling correlation between functional networks and the intricate chunking processes inherent in chess games, encompassing both segmentation and concatenation. It is also worth noting that the functional connectivity network manifests small‐world properties in all conditions. However, among the four conditions considered, only the HD task demonstrated a significant increase in clustering coefficient values and a reduction in path length as compared to the HD task. This discovery underscores the notion that heightened network characteristics are associated with an HD task. These characteristics may indicate an amplified or modified functional network, which aids in the facilitation of perceptual pattern recognition and memory information retrieval across diverse scenarios.

Importantly, the functional connections involving FPC, DFPFC, and SCG also showcased a pronounced association with the club players group's behavioral performance within the HD condition. This implies that club players possess the capability to effectively manage cognitive resources and accurately retrieve pertinent information during the segmentation process, which is able to integrate the information into larger cognitive chunks, ultimately leading to precise and superior moves. Our findings offer evidence that club players are adept at optimizing performance by skillfully modulating the segmentation and connectivity aspects of the chunking process, particularly when faced with distinct levels of difficulty. Consequently, our results serve as substantial support for the pivotal roles of segmentation and concatenation processes employed by club players when confronting challenging conditions. We also believe that this is valuable information that demonstrates significant and objective modulation of brain connectivity in club player group. This highlights the effectiveness of board games as cognitive enhancement tools.

## AUTHOR CONTRIBUTIONS

**Chantat Leong**: Conceptualization; methodology; software; data curation; writing—original draft; visualization; formal analysis; investigation; validation. **Zhiying Zhao**: Supervision; writing—review & editing; formal analysis. **Zhen Yuan**: Funding acquisition; writing—review & editing; supervision; resources. **Bin Liu**: Funding acquisition; writing—review & editing; project administration.

## CONFLICT OF INTEREST STATEMENT

The authors declare no conflicts of interest.

### PEER REVIEW

The peer review history for this article is available at https://publons.com/publon/10.1002/brb3.3488.

## Data Availability

Data substantiating the findings of this study have been made available through Figshare. The Digital Object Identifier (DOI) was: https://doi.org/10.6084/m9.figshare.24211452.v1. The code and more detailed dataset, if required, can be obtained directly from the authors.
